# Evolution of Neuroimaging Findings in Severe COVID-19 Patients with Initial Neurological Impairment: An Observational Study

**DOI:** 10.3390/v14050949

**Published:** 2022-05-01

**Authors:** François Lersy, Caroline Bund, Mathieu Anheim, Mary Mondino, Vincent Noblet, Shirley Lazzara, Clelie Phillipps, Olivier Collange, Walid Oulehri, Paul-Michel Mertes, Julie Helms, Hamid Merdji, Maleka Schenck, Francis Schneider, Julien Pottecher, Céline Giraudeau, Agathe Chammas, François-Daniel Ardellier, Seyyid Baloglu, Khalid Ambarki, Izzie Jacques Namer, Stéphane Kremer

**Affiliations:** 1 Service d’Imagerie 2, Hôpital de Hautepierre, Hôpitaux Universitaires de Strasbourg, 67000 Strasbourg, France; francois.lersy@chru-strasbourg.fr (F.L.); agathe.chammas@chru-strasbourg.fr (A.C.); francois-daniel.ardellier@chru-strasbourg.fr (F.-D.A.); seyyid.baloglu@chru-strasbourg.fr (S.B.); 2 ICANS, Service de Médecine Nucléaire, 67000 Strasbourg, France; c.bund@icans.eu (C.B.); ij.namer@icans.eu (I.J.N.); 3 Engineering Science, Computer Science and Imaging Laboratory (ICube), Integrative Multimodal Imaging in Healthcare, University of Strasbourg-CNRS, UMR 7357, CEDEX, 67000 Strasbourg, France; mondino@unistra.fr (M.M.); vincent.noblet@unistra.fr (V.N.); shirley-ann.lazzara@et.unistra.fr (S.L.); 4 Service de Neurologie, Hôpitaux Universitaires de Strasbourg, 1 Avenue Molière, CEDEX, 67200 Strasbourg, France; mathieu.anheim@chru-strasbourg.fr (M.A.); clelie.phillipps@chru-strasbourg.fr (C.P.); 5 Institut de Génétique et de Biologie Moléculaire et Cellulaire (IGBMC), INSERM-U964/CNRS-UMR7104/Université de Strasbourg, 67400 Illkirch, France; 6 Fédération de Médecine Translationnelle de Strasbourg (FMTS), UR 3072, Université de Strasbourg, 67000 Strasbourg, France; julien.pottecher@chru-strasbourg.fr; 7 Service d’Anesthésie-Réanimation, Nouvel Hôpital Civil, Hôpitaux Universitaires de Strasbourg, 67000 Strasbourg, France; olivier.collange@chru-strasbourg.fr (O.C.); walid.oulehri@chru-strasbourg.fr (W.O.); paul-michel.mertes@chru-strasbourg.fr (P.-M.M.); 8 Service de Médecine Intensive-Réanimation, Nouvel Hôpital Civil, Hôpitaux Universitaires de Strasbourg, 67000 Strasbourg, France; julie.helms@chru-strasbourg.fr (J.H.); hamid.merdji@chru-strasbourg.fr (H.M.); 9 INSERM (French National Institute of Health and Medical Research), UMR 1260, Regenerative Nanomedicine (RNM), CRBS (Centre de Recherche en Biomédecine de Strasbourg), FMTS (Fédération de Médecine Translationnelle de Strasbourg), Faculty of Medicine, University of Strasbourg, 67000 Strasbourg, France; 10 Service de Médecine Intensive Réanimation, Hôpitaux Universitaires de Strasbourg, Hautepierre, 67000 Strasbourg, France; maleka.schenck@chru-strasbourg.fr (M.S.); francis.schneider@chru-strasbourg.fr (F.S.); 11 Service d’Anesthésie-Réanimation et Médecine Péri-Opératoire, Hôpital de Hautepierre, Hôpitaux Universitaires de Strasbourg, 67000 Strasbourg, France; 12 Department of Radiology, IHU Strasbourg, 67000 Strasbourg, France; celine.giraudeau@ihu-strasbourg.eu; 13 Siemens Healthcare, Siemens Healthcare SAS, 67200 Saint Denis, France; khalid.ambarki@siemens-healthineers.com

**Keywords:** COVID-19, neuroimaging, follow-up

## Abstract

Background and Objectives: Cerebral complications related to the COVID-19 were documented by brain MRIs during the acute phase. The purpose of the present study was to describe the evolution of these neuroimaging findings (MRI and FDG-PET/CT) and describe the neurocognitive outcomes of these patients. Methods: During the first wave of the COVID-19 outbreak between 1 March and 31 May 2020, 112 consecutive COVID-19 patients with neurologic manifestations underwent a brain MRI at Strasbourg University hospitals. After recovery, during follow-up, of these 112 patients, 31 (initially hospitalized in intensive care units) underwent additional imaging studies (at least one brain MRI). Results: Twenty-three men (74%) and eight women (26%) with a mean age of 61 years (range: 18–79) were included. Leptomeningeal enhancement, diffuse brain microhemorrhages, acute ischemic strokes, suspicion of cerebral vasculitis, and acute inflammatory demyelinating lesions were described on the initial brain MRIs. During follow-up, the evolution of the leptomeningeal enhancement was discordant, and the cerebral microhemorrhages were stable. We observed normalization of the vessel walls in all patients suspected of cerebral vasculitis. Four patients (13%) demonstrated new complications during follow-up (ischemic strokes, hypoglossal neuritis, marked increase in the white matter FLAIR hyperintensities with presumed vascular origin, and one suspected case of cerebral vasculitis). Concerning the grey matter volumetry, we observed a loss of volume of 3.2% during an average period of approximately five months. During follow-up, the more frequent FDG-PET/CT findings were hypometabolism in temporal and insular regions. Conclusion: A minority of initially severe COVID-19 patients demonstrated new complications on their brain MRIs during follow-up after recovery.

## 1. Introduction

Since the beginning of the COVID-19 outbreak, some cerebral complications related to COVID-19 were documented by brain MRIs [1,2,3,4] and included a wide range of lesions, such as ischemic strokes, hemorrhages, leptomeningeal enhancement, encephalopathy/encephalitis, and perfusion disorders. Besides, some complications, such as cerebral vasculitis [5,6], were suspected on initial MRIs but could not be confirmed thereafter.

Patients with brain fluorodeoxyglucose (FDG) positron emission tomography (PET)/computed tomography (CT) (FDG-PET/CT) abnormalities were also reported [7,8], particularly with bilateral frontal hypometabolism.

The underlying mechanisms involved are probably numerous and non-mutually exclusive [9]. A direct viral cytopathic effect appears to be uncommon, but parainfectious or postinfectious immune-mediated mechanisms may be at work. However, systemic mechanisms of neurological damage, either related to the patient’s critical condition (severe hypoxemia, systemic hypotension) or to cytokine release syndrome or hypercoagulable state, should not be ignored.

Much has been reported over the past year concerning these neuroimaging findings, but little is known about these patients’ natural history and mid-term sequelae. Moreover, data about neurocognitive decline after recovery from COVID-19 are accumulating [10,11], but before our study, to our knowledge, no comparison with neuroimaging findings was performed in a large cohort.

This single-center study conducted in a well-characterized cohort of COVID-19 patients who underwent a brain MRI for neurological symptoms during the acute phase and carried out additional imaging studies during follow-up, after recovery from SARS-CoV-2 infection, was designed to address this issue.

The paper aims at (a) describing the evolution of these neuroimaging findings (MRI and FDG-PET/CT) in patients who recovered from COVID-19 and (b) assessing the neurocognitive outcomes of these patients.

## 2. Material and Methods

This observational and purely descriptive study was approved by the ethical committee of Strasbourg University Hospital (CE-2020-37) and was conducted in accordance with the 1964 Helsinki Declaration and its later amendments.

### 2.1. Patient Cohort and Study Design

During the COVID-19 outbreak between 1 March and 31 May 2020, 112 consecutive COVID-19 patients with neurologic symptoms underwent a brain MRI at Strasbourg University hospitals. Out of these 112 patients, 31 patients underwent additional imaging studies (requested by the physicians in charge of the patient, at least one brain MRI) either at three and/or six months and were finally recruited. These neuroimaging examinations were either carried out systematically or because of a persistent complaint.

Inclusions ended 31 November 2020.

All these 31 patients were initially hospitalized in intensive care units (ICU) for severe disease.

Initially, the most frequent neurological manifestations were:

- Pathological wakefulness when sedative therapies were stopped;
- Delirium;
- Signs of corticospinal tract involvement.

### 2.2. Brain MRIs—Protocols and Interpretation

Imaging studies were conducted either on a 1.5-T MRI or a 3-T MRI. The most frequent sequences performed were 3D T1-weighted spin-echo MRI with and without contrast enhancement; diffusion-weighted, perfusion-weighted, and susceptibility-weighted imaging; and 2D or 3D FLAIR before and after administration of gadolinium-based contrast agent. During follow-up, some of them (PCASL, 3D FLAIR, SWI-Wave-CAIPI) were prototype sequences. 

Brain MRIs were retrospectively reviewed by two neuroradiologists (S.K., and F.L. with 20 and 9 years of experience in neuroradiology, respectively) who reached a consensus concerning the final diagnosis.

### 2.3. Brain Volumetry

Brain tissue volume, including separate estimates of grey matter (GM) and white matter (WM) volumes, normalized for subject head size, was estimated with SIENAX [12], part of FSL [13].

### 2.4. FDG-PET/CT Protocols and Interpretation

PET examinations were performed on a Siemens Vision. 18F-FDG was injected intravenously at 2 MBq/kg, after at least six hours of fasting (except for ad libitum water intake), with capillary glycemia lower than 6.6 mmol/L. Image acquisition was initiated 30 min after 18F-FDG injection, including a low-dose non-contrast transmission CT scan followed by a PET scan with an acquisition time of 10 min. PET data were reconstructed with and without CT-based attenuation correction (TrueX+TOF, ten iterations, five subsets, zoom 2, matrix 880, Gaussian filter 2).

PET FDG were reviewed by two nuclear medicine physicians (IJN and CB). There were two types of analysis: qualitative and semi-quantitative analysis to study colliculi’s metabolism. SUVmax on colliculi were measured and normalized using a ROI on the mesencephalon.

### 2.5. Neurocognitive Assessment

Sixteen patients were seen by two trained neuropsychologists between three and six months after recovery of COVID-19. The results of four patients were not included in the analysis because they were not native French speakers. The remaining 12 patients underwent an evaluation of global cognitive efficiency (mini mental state examination), memory (5-words test and 5-figures test), executive functions (frontal assessment battery (FAB), digit span, fluency), and instrumental functions (Mahieux praxis scale, DO40, Rey’s Figure). 

### 2.6. Statistical Analysis

Data were described using frequency and proportion (n, %) for categorical variables, using mean and standard deviation (SD) for quantitative data. Quantitative data were compared using Mann–Whitney–Wilcoxon testing. A *p*-value lower than 0.05 was considered significant.

## 3. Results

**Figure 1:**
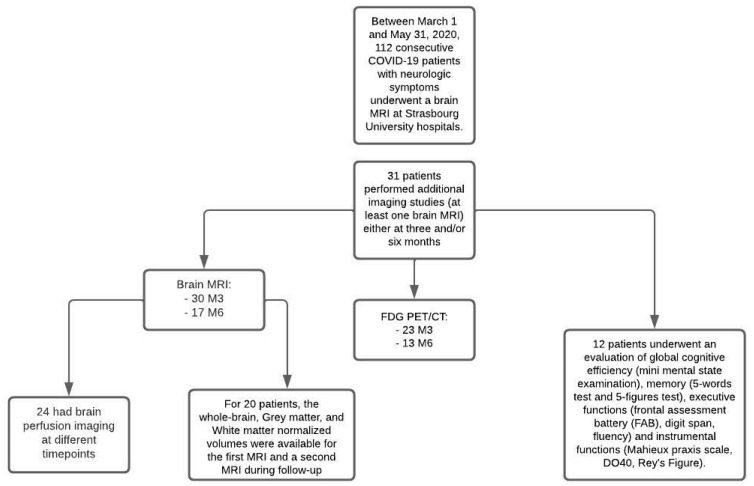
Flow diagram.

Thirty-one patients were finally included in this study (Figure 1): 23 men (74%) and eight women (26%), with a mean age of 61 years (SD ± 12.4; range: 18–79). These patients were hospitalized in ICUs and presented neurological symptoms during France’s first wave of the COVID-19 pandemic. The average length of stay in the ICUs was 30 days (SD ± 31; range: 3–139), and the mean hospital length of stay was 57 days (SD ± 60; range: 12–268).

### 3.1. Brain MRI Findings

Out of the 31 patients, all underwent at least one complementary brain MRI during follow-up: 30 (97%) at three months and 17 (55%) at six months. Overall, 16 (52%) patients underwent three brain MRIs (Table 1).

The first follow-up MRI was performed on average 95 days (SD ± 13; range: 76–132) after the first brain MRI performed during ICU stay, and the second follow-up MRI was realized, when performed, on average 189 days (SD ± 16; range:169–224) after the initial exploration.

(a) Initial brain MRI findings

Brain MRIs were considered normal, unrelated to the current acute clinical presentation, in 10 (32%) cases. Among the 21 (68%) patients with pathological brain MRIs, the neuroimaging findings were:

- Fourteen (45%) cases of focal (single focus or multiple foci) leptomeningeal enhancement (LME);
- Nine (29%) diffuse brain microhemorrhages, which predominantly involved the corpus callosum, the subtentorial juxtacortical WM, the internal capsule, the brainstem, the middle cerebellar peduncles, and the cerebellum, leading to the diagnosis of critical-illness associated cerebral microbleeds (CIAM) [14];
- Four (13%) acute ischemic strokes (acute small vessel infarcts or borderline infarction);
- Four (13%) patients presented with arterial vessel wall thickening displaying homogeneous and concentric enhancement, compatible with cerebral vasculitis [5,6];
- Three (10%) patients had acute inflammatory demyelinating lesions (radiological acute disseminated encephalomyelitis (ADEM) [15] or radiological acute hemorrhagic leukoencephalitis (AHL) [16]).

Patients could have had more than one pattern of lesions.

(b) Evolution of initial neuroimaging findings

The evolution was dissociated in the 14 patients with LME: stability in 3 cases (21%), partial regression in 6 cases (43%), and complete regression in 5 cases (36%).

We noted stability over time of the cerebral microbleeds (CIAM).

**Figure 2:**
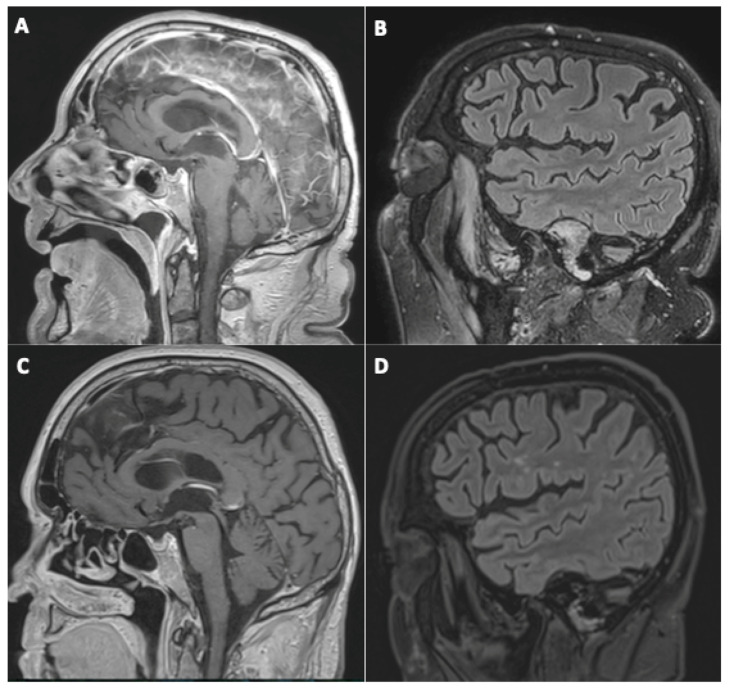
A 79-year-old man (#21) initially hospitalized (ICU) (**A**,**B**), who underwent a second MRI during follow-up 112 days later (**C**,**D**). Sagittal post-contrast three-dimensional T1-weighted spin-echo MR imaging (**A**,**C**) and sagittal FLAIR MR images (**B**,**D**). Basilar artery wall enhancement (**A**) with normalization of the vessel wall imaging during follow-up (**C**), and appearance of white matter FLAIR hyperintensities, presumed of a vascular origin (**D**).

All of the four patients presenting initially with suspected cerebral vasculitis demonstrated normalization of their vessel wall imaging (Figure 2).

For the three patients with acute demyelinating lesions, we noted a sequelae evolution of them.

(c) New findings during follow-up

One patient (3%) experienced ischemic complications: a large-vessel stroke on the first follow-up MRI and a small vessel infarct on the second MRI.

Among the four patients with suspected cerebral vasculitis on the first MRI, one demonstrated a marked increase in WM FLAIR hyperintensities, presumed of a vascular origin (Figure 2).

**Figure 3:**
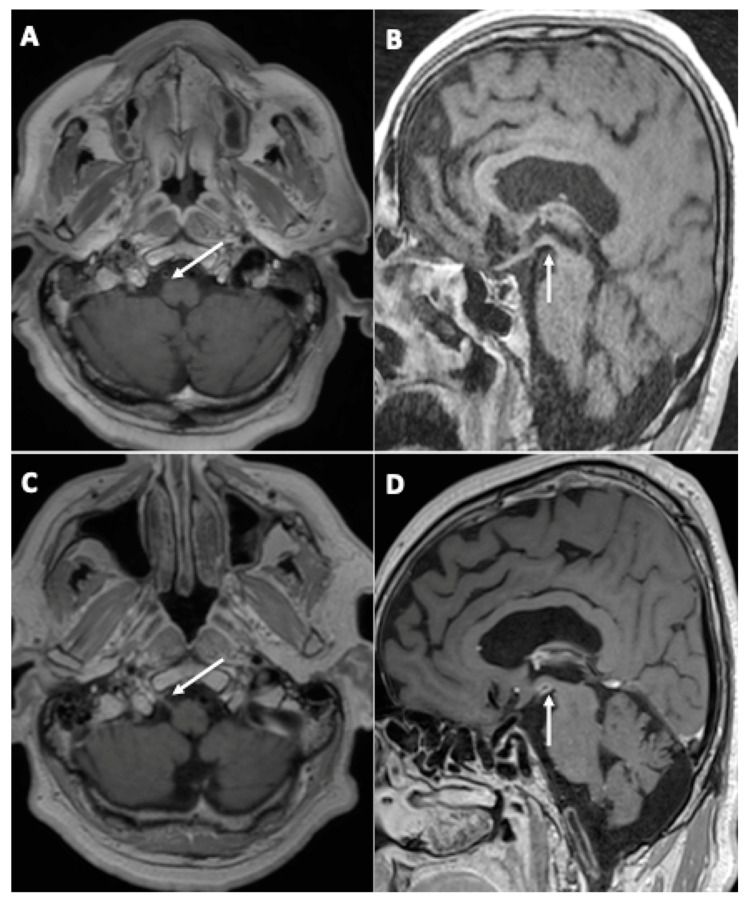
A 67-year-old man (#20) (**A**,**C**) and a 61-year-old man (#7) (**B**,**D**), both initially hospitalized (ICU), who each underwent a second MRI during follow-up 112 (**A**,**C**) and 99 (**B**,**D**) days later, respectively. Axial (**A**,**C**) and sagittal (**B**,**D**) post-contrast three-dimensional T1 weighted spin-echo MR imaging. Appearance of contrast enhancement affecting the right hypoglossal nerve (**C**) (neuritis), and the wall of the posterior cerebral arteries suggestive of cerebral vasculitis.

One patient (3%) demonstrated the appearance of contrast enhancement in the walls of large arteries suggestive of cerebral vasculitis (Figure 3).

Another patient (3%) was diagnosed with right hypoglossal neuritis (Figure 3).

(d) Evolution of perfusion imaging

**Figure 4:**
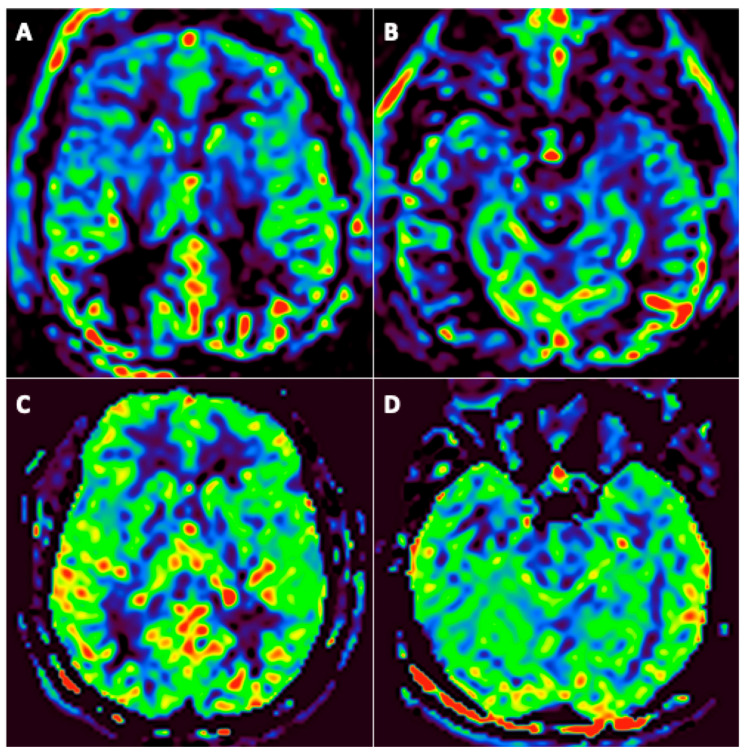
A 46-year-old man (#6) initially hospitalized in the ICU (**A**,**B**), who underwent a second MRI during follow-up 92 days later (**C**,**D**). Axial arterial spin labeling (ASL) brain perfusion imaging (**A**–**D**). Abnormal brain perfusion on initial imaging with frontotemporal hypoperfusion (**A**,**B**) and normalization during follow-up (**C**,**D**).

Among the 31 patients, 24 underwent at least two assessable arterial spin labeling (ASL) brain perfusion imaging at different timepoints, none of them with an intracranial arterial occlusion on MR-angiography. Among these 24 patients, 19 (79%) had abnormal brain perfusion on the initial imaging: 16 (66.7%) patients had hypoperfusion (Figure 4) and 4 (17%) had hyperperfusion. Among these 19 patients, brain perfusion had normalized in 11 (58%) of them by the last imaging session; 1 (5%) still had hyperperfusion; 1 (5%) with initial hyperperfusion had hypoperfusion in the last imaging session; and 6 (37%) patients still had hypoperfusion in the last imaging session. A qualitative improvement in brain perfusion was nevertheless present in 5 (83%) of these 6 patients. Overall, 16 (84%) patients showed at least partial normalization of their brain perfusion.

### 3.2. Brain Volumetry Changes during Follow-Up

The results are presented in Table 2. For 20 patients, the normalized whole-brain, GM, and WM volumes were available from the first MRI and a second MRI during follow-up, and the values were in accordance with the data reported in the literature [17]. The lack of a 3D T1-weighted sequence was the main reason for the missing data. Among these 20 patients, 5 of them started corticosteroid therapy during the ICU stay, before the first brain MRI.

Those five patients each showed a significant increase in volume during follow-up: whole-brain normalized volume +5.9%, GM normalized volume +1.4%, WM normalized volume +10%. This was expected, since corticosteroids are known to lead to reductions in volume after they are newly started, an effect lasting from weeks to months [18,19]. The brain volumes had probably transiently decreased at the time of the first MRI; that is why we chose to exclude these five patients from the complementary analyses.

For the remaining 15 patients, the whole-brain normalized volume was discreetly decreased (−0.4%), and the evolution was different for the GM (−3.2%) and the WM (+2.3%).

Among these 15 patients, eight (#1, 4, 6, 10, 11, 16, 21, 26) demonstrated significant increases (≥5%) in the WM normalized volume (+8.4%). We compared the renal biomarkers (worst pejorative value in the seven days prior to the first MRI) between these eight patients and the seven others, and we found no significant differences: mean higher blood urea nitrogen, 16.9 versus 17.3 mmol/L (*p*-value = 1); mean higher creatinine 130.9 versus 153.9 µmol/L (*p*-value = 0.86); mean lower eGFR, 68.3 versus 63.4 mL/min/1.73 m² (*p*-value = 0.95).

### 3.3. FDG-PET/CT Findings

Among the 31 patients included, 24 underwent FDG PET/CT:

- One patient had PET in the acute phase;
- Twenty-three patients had PET at three months (among them, 12 underwent a second PET at six months);
- One patient underwent PET at six months.

Table 3 shows PET findings at 3 and 6 months.

**Figure 5:**
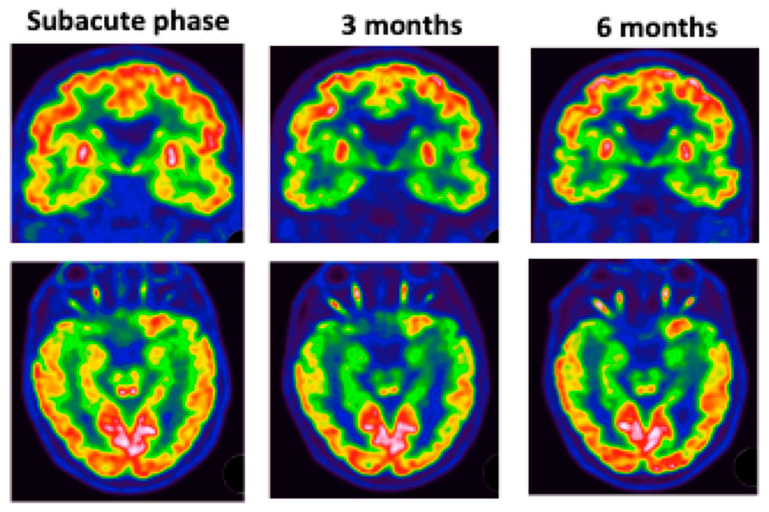
Patient #5: colliculus’s hypermetabolism and left medial temporal hypometabolism at subacute phase. These abnormalities worsened over time.

The most affected regions were the temporal and insular regions (Figure 5).

Thirteen patients had hypermetabolism on colliculi, especially at three months.

Patient #5 (Figure 5) underwent 3 FDG PET scans, which showed hypometabolism in the left intern temporal area and colliculi hyperactivation with worsening of hypo metabolism at 3 months. MRI did not show any abnormalities.

### 3.4. Neurocognitive Assessment

The results are presented in Table 4. All patients had normal global cognitive efficiency. None of them showed fluency impairment, or apraxia or visuospatial constructional disability. The FAB results were normal. Taking the difference between forward and backward digit span as an indicator of a working memory deficit, 6 out of 13 patients had a difference greater than 2.

Although the means of the other tests were within the norms, the performances of some patients were discreetly deficient: 3 out of 13 for verbal memory, 2 out of 13 for visual memory, and 3 of 12 for a denomination task. Planning deficits were observed in 6 of 12 patients.

## 4. Discussion

In this cohort of 31 COVID-19 patients, initially hospitalized in the ICU, who underwent a brain MRI for neurological manifestations, our study showed stability or regression of the lesions in most cases. On conventional imaging, only four patients demonstrated new complications during follow-up.

Twenty-three patients among this cohort underwent FDG PET-CT at three months. Only one patient had no abnormality. Patients had moderate hypometabolisms, especially in temporal regions. Studies often showed frontal hypometabolism, cerebellar hypermetabolism, and diffuse cortical hypometabolism [7,20]. Nevertheless, the inclusion criteria were different, and in our study, we did not have FDG PET-CT performed during the acute phase.

Temporal and insular abnormalities could be age-related. Indeed, in our population we had a mean age of 61 years. Thus, they are difficult to interpret and need to be age-normalized and compared to a healthy group. We could nevertheless mentioned patient #5 (the only one who had a PET at subacute phase) who showed worsening of hypometabolisms.

In 5 (42%) out of 12 patients who underwent a second PET, the abnormalities persisted at six months, and seven (58%) showed improvements. It would be interesting for these patients to redo a FDG PET-CT and to correlate it with new neuropsychological tests.

Concerning colliculi’s hyperperfusion, it could reflect a viral gateway into the central nervous system or direct viral involvement, as discussed in previous studies [21,22]. 

We also presented the evolution of the brain volumetry for 15 COVID-19 patients initially treated in ICU. In order to improve the reliability of these comparisons during follow-up, the same MRI should preferentially be used. If this is not possible, as in our study, normalization to the intracranial volume improves the reproducibility of these measures between different MRIs. A study [23] has demonstrated a mean difference of 0.79% between two MRIs with FSL-SIENAX. We can then assume real modifications of the volumetry during follow-up (GM: −3.2%, WM: +2.3%). Concerning the GM volumetry decrease (loss of volume of 3.2% during an average period of approximately five months), this is much greater than what is expected in healthy adult subjects (loss of volume of about 0.3% per year) [24]. It is known that critically ill patients treated at the ICU can present de novo cerebral atrophy, including loss of GM [25], which may promote neurocognitive decline. The variation in WM volume, especially a decrease at the time of the first MRI, could be due to a state of dehydration [26]. This is likely, since a recent study [27] has shown that among 294 patients treated in ICU, 42.9% were dehydrated upon admission and 54.1% were dehydrated on day 3. Dehydration is a common finding in ICUs that is clinically easily overlooked, and for which the laboratory parameters are insufficient for diagnosis [27].

The evolution of LME, described early in the pandemic on postcontrast FLAIR sequences [1,28], and concordant with a recent neuropathological study that described a meningeal lymphocytic infiltrate in six patients [29], was never studied prior to this study. Even though we observed a declining trend, 9 out of 14 patients (64.3%) still had this abnormality during follow-up. As the circulation of the virus remains very high, we believe that COVID-19 infection should now be included in the differential diagnosis of LME with leptomeningeal metastases and other causes of leptomeningitis [30]. These COVID-19-related LME seem to correspond to an inflammatory process rather than direct viral involvement [9,29].

Concerning the CIAM, the brain microbleeds’ load was stable over time, confirming the regression of the contributing factors, likely indicating deep hypoxemia [14].

Data giving credence to the assumption of cerebral vasculitis as one of the mechanisms triggering COVID-19-induced brain damage are still growing [5,6,31,32].

Our study provides additional arguments in favor of this diagnosis by showing the normalization of identical sequences allowing the study of the vessel wall in four patients. A recent case report [31] has demonstrated the same evolution a few weeks after the first MRI. It is widely known that angiotensin-converting enzyme 2 (ACE2), the main receptor for SARS-CoV-2, is expressed, among others, by endothelial cells [33]. Although a recent pathologic study has shown for the first time that SARS-CoV-2 can infect endothelial cells with diffuse endothelial inflammation [33], the mechanism is not necessarily a direct viral cytopathic effect. However, it may involve other phenomena, such as immune-mediated disorders. This latter assumption is reinforced by the occurrence of a probable fifth case of vasculitis in our cohort during follow-up.

The increase in the WM hyperintensities with presumed vascular origin on FLAIR images, in one patient with suspected vasculitis (1/4, 25%), was, to our knowledge, has never been reported hitherto. In our view, this finding warrants closer monitoring of the different cardiovascular risk factors and may support corticosteroids’ introduction [31]. The true prevalence of vasculitis in this condition remains unknown, especially because dedicated vessel wall imaging is not routinely performed. It seems relevant to carry out these sequences for the patients with common cerebral vasculitis complications, such as acute ischemic stroke, and subarachnoid or cerebral hemorrhages.

One patient demonstrated changes compatible with right hypoglossal neuritis during follow-up, probably immune-mediated, as previously described after SARS-CoV-2 infection [34,35].

These two kinds of post-infectious modifications (vasculitis, neuritis) should make us aware, in the first few weeks following infection with SARS-CoV-2, of the possibility of such aberrant immune responses.

Regarding neuropsychological assessment performed after 3 months, there was no evidence of severe cognitive sequelae following COVID-19. Only working memory and executive functions seemed slightly diminished in nearly half patients. Concerning memory, the tests used in this study present a ceiling effect which does not allow one to finely characterize these deficits or to attempt anatomoclinical correlations. Further studies are needed to further explore these abnormalities.

Even if this cohort of 31 COVID-19 patients remains unique (MRI including volumetry/perfusion imaging, and PET-TDM results), our study has several limitations, mainly due to its retrospective design and the low number of patients included. The main limitation concerns the missing data, both from imaging exams and the neurocognitive assessments.

## Figures and Tables

**Table 1:** Brain MRI changes.

	Sex	Age	Hospital Length of Stay (Days)	ICU Length of Stay (Days)	Neurological Manifestations at the Time of the First MRI	First MRI	Second MRI	Third MRI
#1	F	59	14	10	Pathological wakefulness when sedative therapies were stopped	Focal LME	Unchanged Stability of LME (+79 days)	Unchanged Stabilité of LME (+176 days)
#2	M	62	35	19	Delirium	Diffuse LME	Partial regression of LME(+98 days)	Unchanged Stability of LME (+224 days)
#3	M	60	20	19	Delirium/Clinical signs of corticospinal tract involvement	Normal	Unchanged (+98 days)	Unchanged (+182 days)
#4	M	50	12	9	Delirium	Normal	Unchanged (+97 days)	Unchanged (+174 days)
#5	M	66	46	23	Aphasia/Clinical signs of corticospinal tract involvement	Normal	Unchanged (+77 days)	NR
#6	M	46	20	16	Pathological wakefulness when sedative therapies were stopped	Normal	Unchanged (+92 days)	NR
#7	M	61	24	8	Delirium/Clinical signs of corticospinal tract involvement	Focal LME	Partial regression of LME Appearance of contrast enhancement in the wall of large arteries (+99 days)	NR
#8	F	75	21	11	Delirium	Diffuse LME CIAM	Partial regression of LME Stability of CIAM (+89 days)	Unchanged (+181 days)
#9	F	59	23	9	Confusion	Normal	Unchanged (+84 days)	NR
#10	M	54	55	39	Pathological wakefulness when sedative therapies were stopped	Focal LME CIAM	Partial regression of LME Stability of CIAM (+91 days)	Unchanged (+189 days)
#11	M	66	29	7	Delirium	Suspicion of cerebral vasculitis	Regression of the vessel wall enhancement Otherwise unchanged Stability of the WM FLAIR hyperintensities (+90 days)	NR
#12	F	71	59	27	Pathological wakefulness when sedative therapies were stopped	Normal	Unchanged (+78 days)	NR
#13	F	18	20	7	Confusion	Normal	Unchanged (+76 days)	NR
#14	F	69	51	7	Clinical signs of corticospinal tract involvement /Cerebellar ataxia	Normal	Unchanged (+79 days)	Unchanged (+177 days)
#15	M	57	49	41	Pathological wakefulness when sedative therapies were stopped	Diffuse LME CIAM	Complete regression of LME Stability of CIAM (+94 days)	NR
#16	M	69	72	48	Pathological wakefulness when sedative therapies were stopped	Focal LME CIAM Acute small vessel infarct	Complete regression of LME Stability of CIAM Large-vessel stroke (CPA) (+98 days)	Acute small vessel infarct Otherwise unchanged (+189 days)
#17	M	71	44	33	Delirium	Diffuse LME CIAM	Partial regression of LME Stability of CIAM (+92 days)	Increase in the WM FLAIR hyperintensities presumed of a vascular origin Otherwise unchanged (+169 days)
#18	F	72	38	30	Lower extremity spasticity	Borderzone infarct Focal LME	Unchanged (+105 days)	Unchanged (+202 days)
#19	F	67	65	38	Pathological wakefulness when sedative therapies were stopped/Clinical signs of corticospinal tract involvement	Normal	Unchanged (+105 days)	NR
#20	M	67	46	8	Pathological wakefulness when sedative therapies were stopped	Suspicion of cerebral vasculitis	Regression of the vessel wall enhancement Stability of the WM FLAIR hyperintensities Right hypoglosses neuritis Otherwise unchanged (+112 days)	NR
#21	M	79	73	45	Pathological wakefulness when sedative therapies were stopped	Suspicion of cerebral vasculitis CIAM	Regression of the vessel wall enhancement Increase in the WM FLAIR hyperintensities presumed of a vascular origin Stability of CIAM (+112 days)	NR
#22	M	61	37	28	Pathological wakefulness when sedative therapies were stopped	Suspicion of cerebral vasculitis CIAM	Regression of the vessel wall enhancement Stability of the WM FLAIR hyperintensities (+110 days)	Unchanged (+186 days)
#23	M	35	52	35	Seizures	Normal	Unchanged (+105 days)	Unchanged (+195 days)
#24	M	68	31	18	Pathological wakefulness when sedative therapies were stopped	CIAM	Stability of CIAM (+132 days)	Unchanged (+188 days)
#25	M	60	29	3	Cognitive impairment	Borderzone infarct	Unchanged (+86 days)	NR
#26	M	76	23	6	Delirium	Focal LME Acute small vessel infarct	Stability of LME (+101 days)	Stability of LME (+185 days)
#27	M	52	30	12	Delirium/Clinical signs of corticospinal tract involvement	CIAM	Stability of CIAM (+79 days)	Unchanged (+193 days)
#28	M	67	215	75	Delirium	Focal LME	Complete regression of LME (+92 days)	Unchanged (+174 days)
#29	M	55	198	109	Pathological wakefulness when sedative therapies were stopped	Radiological ADEM Focal LME	Sequellary evolution of the inflammatory lesions Complete regression of LME (+96 days)	NR
#30	M	56	268	139	Pathological wakefulness when sedative therapies were stopped	Radiological AHL Focal LME	Sequellary evolution of the inflammatory lesions Stability of LME (+93 days)	NR
#31	M	73	81	65	Pathological wakefulness when sedative therapies were stopped	Radiological AHL Focal LME	Sequellary evolution of the inflammatory lesions Complete regression of LME (+223 days)	NR

F: female; M: male; NR: not realized.

**Table 2:** Brain volumetry changes.

Patients	Time between First and Last MRI(Days)	Brain Normalized Volume (mL) (*First MRI—Last MRI/Evolution)*	Grey Matter Normalized Volume (mL)(*First MRI—Last MRI/Evolution)*	White Matter Normalized Volume (mL) (*First MRI—Last MRI/Evolution)*
#1	176	1459–1507/+3.3%	725–733/+1.1%	734–774/+5.5%
#3 *	182	1281–1349/+5.3%	602–625/+3.9%	679–724/+6.5%
#4	174	1502–1505/+0.2%	736–697/−5.3%	765–807/+5.5%
#6	92	1481–1552/+4.8%	708–723/+2%	772–829/+7.3%
#7	99	1439–1354/−5.9%	644–604/−6.1%	795–749/−5.8%
#10	189	1349–1405/+4.1%	671–675/+0.5%	677–730/+7.7%
#11	90	1350–1401/+3.8%	643–648/+0.9%	706–753/+6.5%
#15	94	1473–1450/−1.5%	700–674/−3.7%	773–775/+0.4%
#16	189	1313–1341/+2.1%	625–612/−2.1%	688–728/+5.9%
#17	169	1412–1409/−0.2%	616–657/+6.7%	796–752/−5.5%
#18	202	1567–1470/−6.2%	756–689/−8.9%	810–781/−3.6%
#19 *	105	1365–1425/+4.4%	675–674/−0.2%	690–751/+8.9%
#20	112	1357–1359/+0.1%	639–640/+0.2%	718–719/+0.1%
#21	112	1311–1304/−0.5%	669–587/−12.1%	642–716/+11.6%
#22 *	186	1150–1178/+2.4%	534–509/−4.6%	616–668/+8.4%
#23 *	105	1491–1567/+5.1%	729–728/−0.1%	762–839/+10.1%
#25 *	86	1353–1511/+11.6%	647–694/+7.2%	706–817/+15.7%
#26	185	1257–1322/+5.2%	669–618/−7.5%	588–703/+19.6%
#27	193	1572–1494/−5%	765–717/−6.2%	807–776/−3.8%
#29	96	1580–1483/−6.2%	651–625/−4%	928–857/−7.6%
All patients (*n* = 20)	141.8 ± 45	1403–1419/+1.1%	670–656/−2.1%	733–762/+4%
Patients under corticosteroids at the time of the first MRI (*n* = 5)	132.8 ± 47	1328–1406/+5.9%	637–646/+1.4%	691–760/+10%
Patients without corticosteroids (*n* = 15)	144.8 ± 45	1428–1423/−0.4%	681–659/−3.2%	746–763/+2.3%
LME on the first MRI (*n* = 8/15)	149.6 ± 45	1410–1409/−0.1%	663–650/−2%	747–759/+1.6%
CIAM on the first MRI (*n* = 6/15)	157.6 ± 44	1405–1400/−0.4%	674–653/−3.1%	730–746/+2.2%
Acute ischemic stroke on the first MRI (*n* = 3/15)	192 ± 9	1379–1377/−0.1%	683–639/−6.4%	695–737/+6%
Suspicion of cerebral vasculitis on the first MRI (*n* = 3/15)	104.6 ± 13	1339–1354/+1.1%	650–625/−3.8%	688–729/+6%

* Corticosteroid therapy started before the first brain MRI.

**Table 3:** FDG-PET CT findings.

	PET in Acute Phase	PET at 3 Months	PET at 6 Months
#1	NR	NR	No abnormalities
#2	NR	Colliculus’s hypermetabolism; Left medial temporal hypometabolism	Unchanged
#3	NR	Colliculus’s hypermetabolism; Bilateral temporal polar and insular lobes (L > R) hypometabolism	Unchanged
#4	NR	Colliculus’s hypermetabolism; bilateral temporo-insular and right centro-opercular region hypometabolism	Colliculus’s hypermetabolism; Improvement of hypometabolism
#5	Colliculus’s hypermetabolism; Left medial temporal hypometabolism	Colliculus’s hypermetabolism; Left medial temporal hypometabolism	Regression of colliculus’s hypermetabolism; Stability of left medial temporal hypometabolism
#10	NR	Bilateral temporo-insular lobes and middle cerebral artery territories hypometabolism (R > L)	Bilateral medial temporal hypometabolism
#11	NR	Bilateral temporo-insular and parietal lobes hypometabolism	NR
#12	NR	Left medial temporal hypometabolism	NR
#13	NR	Bilateral temporo insular hypometabolism	NR
#14	NR	Left medial temporal hypometabolism	Unchanged
#15	NR	Bilateral temporo insular hypometabolism	NR
#16	NR	Colliculus’s hypermetabolism; Bilateral temporo insular hypometabolism (L > R)	Left medial temporal hypometabolism
#17	NR	Colliculus’s hypermetabolism; Medial temporal and right thalamus hypometabolism	NR
#18	NR	Colliculus’s hypermetabolism	No abnormalities
#19	NR	Bilateral temporal hypometabolism	NR
#20	NR	Colliculus’s hypermetabolism	No abnormalities
#21	NR	Left medial temporal hypometabolism	Unchanged
#22	NR	Colliculus’s hypermetabolism; Bilateral temporal hypometabolism	Unchanged
#23	NR	Colliculus’s hypermetabolism; Bilateral temporal hypometabolism	NR
#24	NR	No abnormalities	No abnormalities
#26	NR	Left medial temporal hypometabolism	NR
#27	NR	Colliculus’s hypermetabolism; Bilateral temporal hypometabolism bitemporal	NR
#28	NR	Left colliculus hypermetabolism;^1Right fronto-temporo-insular and left thalamus hypometabolism	NR
#29	NR	Colliculus’s hypermetabolism; Bilateral parietal and temporal hypometabolism	NR

L: left; NR: not realized; R: right.

**Table 4:** Mean score and standard deviation of each test of the neuropsychological assessment performed between three and six months after recovery of COVID-19.

Tests	Means	Standard-Deviation
**MMSE**	28.23	1.24
**Dubois’ 5-words test**	9.75	0.45
**5-figures test**	9.31	0.75
**Digit span forward**	5.46	0.88
**Digit span backward**	3.92	0.86
**FAB**	17	1.21
**Litteral fluency (1 min)**	18.17	6.45
**Praxis scale (Mahieux)**	7.5	0.52
**DO40**	37.83	2.86
**Categorial fluency (1 min)**	21,5	8.8
**ROCF**	31.71	3.41
**Incomplete letters (VOSP)**	19.08	1.38

DO 40: Oral denomination 40; FAB: Frontal Assessment Battery; MMSE: Mini-Mental State Examination; ROCF: Rey Osterrieth Complex Figure; VOSP: Visual Object and Space Perception Battery.

## Data Availability

We state that the data published are available and anonymized and will be shared upon request by email to the corresponding author from any qualified investigator for purposes of replicating procedures and results.

## Abbreviations

ACE2	angiotensin-converting enzyme 2
ADEM	Acute disseminated encephalomyelitis
AHL	Acute hemorrhagic leukoencephalitis
ASL	Arterial spin labeling
CT	computed tomography
GM	grey matter
FAB	frontal assessment battery
FDG	fluorodeoxyglucose
FDG PET/CT	fluorodeoxyglucose positron emission tomography/computed tomography
ICU	intensive care units
LME	leptomeningeal enhancement
PET	positron emission tomography
SD	standard deviation
WM	white matter
